# Association of *Stmn1* Polymorphism and Cognitive Function: An Observational Study in the Chinese Adults

**DOI:** 10.31083/AP38719

**Published:** 2025-02-28

**Authors:** Hui Ma, Zhengtu Cong, Lijuan Liang, Zhaoxia Su, Jing Zhang, Hua Yang, Man Wang

**Affiliations:** ^1^Psychological Counseling and Treatment Center, Hainan Provincial Anning Hospital, 570207 Haikou, Hainan, China; ^2^Department of Clinical Psychology, The First Affiliated Hospital of Hainan Medical University, 570102 Haikou, Hainan, China; ^3^Department of Clinical Psychology, Hainan Pingshan Hospital, 572299 Wuzhishan, Hainan, China; ^4^The Seventh Department of Psychiatry, Hainan Provincial Anning Hospital, 570207 Haikou, Hainan, China; ^5^Department of Clinical Psychology, The 2nd Clinical Medical College of Jinan University, Shenzhen People’s Hospital, 518020 Shenzhen, Guangdong, China

**Keywords:** *Stmn1*, gene polymorphism, cognitive function, Stroop Color-Word Test, Hopkins Verbal Learning Test

## Abstract

**Background:**

Stathmin1 (Stmn1) is a protein highly expressed during the development of the central nervous system. The phosphorylation of Stmn1 involves microtubule dynamics, so Stmn1 plays a vital part in neurite outgrowth and synaptic plasticity. Previous studies reported that *Stmn1* genetic variants influence fear and anxiety as well as cognitive-affective processing. However, no study reported on the relationship between *Stmn1* gene polymorphism and cognition in Chinese. Thus, this association was investigated in the present study.

**Methods:**

A total of 129 healthy Han Chinese were genotyped for *Stmn1* rs182455 polymorphism by polymerase chain reaction and restriction fragment length polymorphism analyses. Cognitive function was assessed using the Stroop Color-Word Test (SCWT) and Hopkins Verbal Learning Test-Revised (HVLT-R).

**Results:**

In the present sample, rs182455 CC, CT, and TT genotypes were found in 56 (43.41%), 65 (50.39%) and 8 (6.20%) cases, respectively. The genotype distribution did not deviate from Hardy-Weinberg equilibrium (χ^2^ = 3.715, *p* = 0.054). Significant differences were found between the three rs182455 genotypes and between the CC and (CT+TT) genotype groups in the Stroop Color (SC) scores of the SCWT (F = 3.322, 2.377; *p *= 0.039, 0.019, respectively) and the total recall (TR) scores on the HVLT-R (F = 3.118, 2.225; *p* = 0.048, 0.028, respectively). There was a female-specific difference in SC scores between the three rs182455 genotypes (F = 2.318, *p* = 0.023). The rs182455 genotype distribution showed no significant difference between two sexes (χ^2^ = 1.313, *p * = 0.519), whereas significant differences were seen in SC and TR scores between two sexes (t = –2.294, –2.490; *p* = 0.023, 0.014, respectively).

**Conclusions:**

The findings suggest that rs182455 *Stmn1* polymorphism might affect cognitive flexibility and immediate free recall in healthy Chinese individuals, especially females.

**Main Points**

1. The number of CC, CT, and TT rs182455 polymorphism genotypes was 56 (43.41%), 65 (50.39%) and 8 (6.20%), respectively, which did not deviate from Hardy-Weinberg equilibrium.

2. Significant differences were found between the three rs182455 genotypes and the CC and (CT+TT) genotype groups in Stroop Color (SC) scores on the Stroop Color-Word Test (SCWT) and total recall (TR) scores on the Hopkins Verbal Learning Test-Revised (HVLT-R).

3. Female-specific differences in SC scores were found between the three rs182455 genotypes.

## 1. Introduction

The human brain receives input from the outside world and converts it to internal psychological activity to control individual behaviors; this is the cognitive process [1]. Cognition, which is the most basic human psychological process, refers to acquiring knowledge, applying knowledge, or processing information. Cognition is critical to normal psychological function and cognitive impairment is the core symptom of many disorders, including neurodegenerative (e.g., Alzheimer’s disease (AD)) [2, 3, 4] and psychiatric diseases (e.g., schizophrenia and affective disorders) [4, 5]. Therefore, it is important to investigate the factors that contribute to cognition.

Intelligence is a general cognitive ability and a quantitative trait that accounts for much of the variation in diverse cognitive abilities. Previous studies demonstrated that general cognitive ability is approximately 50%–70% heritable [6, 7], meaning that genetic differences account for more than 50% of individual variation in performance on tests of cognitive ability. In contrast, the influence of the environment on general cognitive ability is less significant and unstable [8]. Twin studies and molecular genetic studies of unrelated persons have both converged on similar heritability estimates [9, 10]. Cognitive functions other than intelligence include diverse abilities, such as reasoning, memory, selective attention, processing speed, mental rotation, knowledge, and executive function. However, less research has been conducted on factors related to cognitive functions beyond intelligence.

In recent decades, the assessment of cognitive functions has gained increasing widespread attention. Previous studies focused on the effect of *Stathmin1* (*Stmn1)* polymorphisms on individual emotion regulation, whereas the present study planned to evaluate an individual’s ability to inhibit a habitual response and react selectively to information, as well as complicated verbal learning and memory, which is different from and more multifaceted than previous studies. The Stroop Color-Word Test (SCWT) is a frequently used test that measures an individual’s ability to inhibit the normal response in favor of an unexpected response [11]. Previous studies have revealed the functions of the SCWT to assess cognitive abilities including selective attention, processing speed, working memory, and cognitive flexibility [12, 13]. In the present study, the SCWT was used to evaluate the participants’ ability to inhibit a habitual response and react selectively to information. The Hopkins Verbal Learning Test-Revised (HVLT-R) is a multicomponent wordlist learning task developed to assess verbal learning and memory [14], which are important aspects of cognitive function. The utility and validity of the HVLT-R have been demonstrated in individuals with a wide range of different races and ethnicities with significant cognitive impairments, such as AD [15, 16, 17] and Huntington’s disease [18]. It also has high ability of classification accuracy for distinguishing individuals with dementia from those without dementia [15, 19] and patients with AD from those with vascular dementia [17, 20]. Therefore, the HVLT-R was used to evaluate verbal learning and memory function for the participants in the present study.

*Stmn1* is highly expressed in central nervous system brain regions, such as the amygdala, prefrontal cortex, and nucleus accumbens, in both mice and humans [4, 21, 22]. It is involved in microtubule (MT) dynamics and neuronal growth and, thus, plays a vital part in neurite outgrowth and synaptic plasticity [23]. Synaptic plasticity refers to the property that the connection strength and functional properties of synapses can change. This plasticity is the basis for the ability of the nervous system to adapt to environmental changes and to execute cognitive functions such as learning and memory. Synaptic plasticity mainly includes short-term and long-term types. Long-term potentiation (LTP) is an important manifestation of long-term synaptic plasticity, which refers to the phenomenon of persistent enhancement of synaptic transmission efficiency and strength that occurs when synapses between two neurons are subjected to a certain form of stimulation. Currently, memory is thought to be encoded by changes in synaptic strength. Thus, LTP plays a crucial role in the process of learning to remember. However, the biological mechanism of LTP has not been fully elucidated. Animal studies found that *Stmn1* gene knockout mice showed deficits in spike-timing-dependent LTP, and exhibited deficient memory related to fear conditioning and the recognition of danger in innately aversive environments, relative to wildtype controls [21].

Based on the involvement of *Stmn1* in LTP and memory, it was hypothesized that *Stmn1* variation primarily impacts human cognition. Brocke *et al*. [24] demonstrated the significant impact of two single nucleotide polymorphisms (SNPs) of the *Stmn1* gene (rs182455 and rs213641) on fear and anxiety responses in healthy German volunteers. Ehlis *et al*. [23] demonstrated the effect of the rs182455 SNP on cognitive-affective processing in healthy German volunteers. Previous research results on the effect of *Stmn1* on human emotion regulation and executive function indicated that *Stmn1* genes and proteins may influence human cognitive function. However, no study has reported a correlation between *Stmn1* gene polymorphisms and cognitive function assessed by the SCWT or HVLT-R. SNP rs182455 is located within or close to the putative transcriptional control region and affects the Stmn1 protein expression level. Informed by the two studies above [23, 24], the present study explored the correlation of *Stmn1* SNP rs182455 with cognitive function in a healthy Chinese population.

## 2. Materials and Methods

### 2.1 Participants 

On the basis of the research target and type, experimental design, resource constraints, etc., we used G*Power 3.0 (Düsseldorf, North Rhine-Westphalia, Germany), a widely used statistical software [25], to calculate the present sample size in the experimental design phase (Test family: F tests. Statistical test: One-way analysis of variance (ANOVA), fixed effects, omnibus, one-way. Input Parameters: Type I error, α = 0.05; Type II error, β = 0.20; Effect size f = 0.30. Output Parameters: Total sample size = 111). In the present study, we employed stratified random sampling methodology. Firstly, the sample was stratified on the basis of sex, resulting in the overall sample divided into two separate strata: males and females. The sub-sample size was estimated within each stratum, primarily based on the overall sample size and the proportion of males and females in the natural population. The ratio between the two sex strata was approximately 1:1. Subsequently, among the volunteers who met the enrollment criteria, random sampling was conducted within each stratum with the assistance of a random number table. Ultimately, the sub-samples drawn from both strata were combined to form the final overall sample. A total of 136 healthy volunteers were recruited from a university in China by random stratified sampling. Seven volunteers who failed to complete the neurocognitive assessment were excluded. Thus, 129 volunteers were identified for the present study. The inclusion criteria included (1) being Chinese Han, (2) being 18–30 years old, (3) having ability to complete the neurocognitive assessments, and (4) having physical and mental health. The exclusion criteria included (1) a history of neurological, psychiatric, or severe physical illness, (2) having neurological, psychiatric, or severe physical illness, (3) being younger than 18 or more than 30 years old, (4) not able to complete the neurocognitive assessment, and (5) having a relative(s) also enrolled in the present study. Each volunteer was interviewed by a psychiatrist for enrollment. Each enrolled subject provided written informed consent. The Ethics Committee of Hainan Provincial Anning Hospital approved this study (No.202206).

### 2.2 Neurocognitive Assessment

In 1935, Stroop [26] introduced the SCWT for the first time. Since then, the version developed by Golden & Freshwater (2002) has become the most popular [11]. In SCWT, Chinese characters are utilized as test materials, and the reaction time becomes an index to reproduce the Stroop effect because of contradictory words and colors. The SCWT consists of three pages: the first for words, the second for colors, and the last for color–words. The participants are instructed to complete the SCWT as quickly as possible during a 45-s period for each page [11]. For the first page, the participant is instructed to read the names of colors printed in black ink (W); this part is believed to represent simple reading and may be influenced by speech/motor problems or learning disabilities [27]. For the second page, the participant is asked to name the color (C) of the X symbol; this might be impaired by speech/motor function or color blindness. For the last page, each color-word is printed in a contrasting color (e.g., the word “red” is printed in blue ink), and the participant is asked to name the color of the ink, not the word, as quickly as possible. This task is assumed to measure both cognitive flexibility and the ability to inhibit the usual response [28]. The three WCST variables (subscores) are the scores of the Stroop Word (SW), Stroop Color (SC), and Stroop Color-Word (SCW) tests.

The HVLT-R developed by Benedict *et al*. [14] is an extension of the HVLT [29]. It consists of 12 nouns, with four words drawn from each of three semantic categories. The participant reads the list at a rate of one word per 2 s, and then attempts to freely recall as many words as they can remember in any order. Correct responses range from 0 to 12, with no deduction for repeated words. The trial involves the presentation of the word list and then free recall is repeated twice, yielding total immediate recall scores ranging from 0 to 36. The delayed recall component occurs after a delay of 20–25 min (occupied by non-verbal tasks). The participant is asked to recall the same list of words without the benefit of having them presented before the recall. The delayed recognition trial immediately follows, in which a list of 24 words is read. This list includes 12 words from the recall list and 12 false positives, half of which are semantically related to the recall list and half are not. The participant must state if each word appeared on the original list. The HVLT-R is composed of three free recall learning trials, a delayed recall trial, and a recognition task [30]. The four HVLT-R variables (subscores) are the scores of total recall (TR), delayed recall (DR), true positives (TPs), and false positives (FPs).

### 2.3 Genotyping

Each subject had a 2 mL venous blood drawn for genotyping. Genomic DNA was extracted by the standard potassium iodide method. Polymerase chain reaction was used to amplify gene fragments containing *Stmn1* SNP rs182455, and restriction fragment length polymorphism analysis was used for subsequent genotyping, which was carried out as described in our previous studies [31, 32, 33]. The rs182455 genotype of each subject was identified independently by two different analysts. When the results of the two experimenters were inconsistent, the experiments were repeated separately until the results were consistent.

### 2.4 Statistical Analysis

The data are presented as the percent frequency or the mean and standard deviation (SD). The chi-squared test was used to assess genotype distribution and Hardy-Weinberg equilibrium (HWE). One-way analysis of variance (ANOVA) was used to compare the mean SCWT and HVLT-R scores between the three genotype groups. The independent sample *t-*test was used to compare the mean age, education years, and SCWT and HVLT-R scores between the male and female participants, and SCWT and HVLT-R scores between the two dominant model genotype groups or two recessive model genotype groups. A *p*-value < 0.05 was set as the level of statistical significance. The statistical software, SPSS 22.0 for Windows (SPSS Inc, Chicago, IL, USA), was used to conduct all statistical analyses.

## 3. Results

### 3.1 Sample Characteristics

A total of 129 volunteers (57 males, 44.2%; 72 females, 55.8%) were identified as the experimental subjects for the present study. The ages ranged from 20 to 26 years, with a mean age ± standard deviation (SD) of 22.98 ± 1.57 years. The education years ranged from 14 to 22 years, with a mean ± SD of 17.16 ± 1.76 years. No significant differences were found in age (t = 0.576, *p* = 0.566) or education years (t = 1.299, *p* = 0.196) between the male and female participants.

### 3.2 HWE Results 

The genotype counts and frequencies for SNP rs182455 in the total sample are presented in Table 1. The numbers of CC, CT, and TT rs182455 genotypes were 56 (43.41%), 65 (50.39%) and 8 (6.20%), respectively. The genotype distribution was not demonstrated to deviate significantly from HWE (χ^2^ = 3.715, *p *= 0.054). Moreover, the distribution was not significantly different from that of previous samples of 160 Asian individuals (T = 0.344, χ^2^ = 0.573, *p* = 0.449) and 102 East Asian individuals (T = 0.382, χ^2^ = 2.361, *p* = 0.124) (https://www.ncbi.nlm.nih.gov/snp/rs182455). Therefore, we can consider the sample in the present study to be representative of the general Chinese population.

**Table 1.** **Effects of *Stmn1* gene polymorphism on SCWT and HVLT-R scores**.

SNP	Genotype	Total N (%)	SCWT			HVLT-R			
			SW	SC	SCW	TR	DR	TP	FP
rs182455	CC	56 (43.41%)	106.14 ± 14.83	79.66 ± 13.36	48.27 ± 8.38	28.45 ± 4.05	10.23 ± 1.74	11.75 ± 0.58	0.52 ± 0.71
	CT	65 (50.39%)	105.65 ± 15.39	74.40 ± 14.48	46.20 ± 10.04	26.75 ± 3.66	9.60 ± 1.92	11.49 ± 0.71	0.51 ± 0.89
	TT	8 (6.20%)	110.38 ± 17.56	69.25 ± 10.32	44.25 ± 9.51	28.38 ± 3.58	10.00 ± 1.31	11.88 ± 0.35	0.25 ± 0.46
	*F*		0.341	3.322	1.105	3.118	1.844	3.074	0.411
	*p *value		0.711	0.039	0.334	0.048	0.162	0.050	0.664
Dominant model	CC	56 (43.41%)	106.14 ± 14.83	79.66 ± 13.36	48.27 ± 8.38	28.45 ± 4.05	10.23 ± 1.74	11.75 ± 0.58	0.52 ± 0.71
	CT+TT	73 (56.59%)	106.16 ± 15.58	73.84 ± 14.12	45.99 ± 9.94	26.93 ± 3.66	9.64 ± 1.86	11.53 ± 0.69	0.48 ± 0.85
	*t*		–0.008	2.377	1.382	2.225	1.833	1.929	0.272
	*p *value		0.994	0.019	0.169	0.028	0.069	0.056	0.786
Recessive model	CT+CC	121 (93.80%)	105.88 ± 15.07	76.83 ± 14.16	47.16 ± 9.33	27.54 ± 3.92	9.89 ± 1.86	11.61 ± 0.66	0.51 ± 0.81
	TT	8 (6.20%)	110.38 ± 17.56	69.25 ± 10.32	44.25 ± 9.51	28.38 ± 3.58	10.00 ± 1.31	11.88 ± 0.35	0.25 ± 0.46
	*t*		–0.810	1.487	0.853	–0.588	–0.161	–1.898	0.907
	*p *value		0.419	0.140	0.395	0.557	0.873	0.085	0.366

Abbreviations: SCWT, Stroop Color-Word Test; HVLT-R, Hopkins Verbal Learning Test-Revised; SW, Stroop Word; SC, Stroop Color; SCW, Stroop Color-Word; TR, total recall; DR, delayed recall; TP, true positive; FP, false positive. The SCWT and HVLT-R data are presented as the means ± standard deviation. The three WCST variables are scores of SW, SC and SCW, and the four HVLT-R variables are scores of TR, DR, TP and FP.

### 3.3 Relationship between Stmn1 Polymorphism and Cognitive Function in the Overall Sample 

The SCWT and HVLT-R scores are presented in Table 1. The three rs182455 genotype groups (CC, CT, TT) showed significant differences in SC scores on the SCWT (F = 3.322, *p *= 0.039) and TR scores of the HVLT-R (F = 3.118, *p* = 0.048) as follows: TT < CT < CC for SC, and CT < TT < CC for TR. However, no significant differences were observed for the SW or SCW scores on the SCWT or DR, TP, or FP scores on the HVLT-R among the different genotype groups (all *p* > 0.05).

Subsequently, we converted the three genotype groups to two recessive model groups and two dominant model groups. Significant differences were demonstrated in SC scores on the SCWT (F = 2.377, *p* = 0.019) and TR scores on the HVLT-R (F = 2.225, *p* = 0.028) between the CC and (CT+TT) genotype groups (Table 1). In contrast, no significant differences were found for any SCWT or HVLT-R scores between the (CT+CC) and TT genotype groups (Table 1, all *p* > 0.05).

### 3.4 Relationship between Stmn1 Polymorphism and Cognitive Function in Male and Female Participants 

Differences in SCWT and HVLT-R scores among the rs182455 genotype groups were analyzed separately by sex. Significant female-specific differences were found in SC scores on the SCWT among those with rs182455 genotypes (Table 2, F = 2.318, *p* = 0.023). However, no significant male-specific differences were found in any SCWT or HVLT-R scores (Table 3, all *p* > 0.05).

**Table 2.** **Effects of* Stmn1* gene polymorphism on SCWT and HVLT-R scores in females**.

SNP	Genotype	Total N (%)	SCWT			HVLT-R			
			SW	SC	SCW	TR	DR	TP	FP
rs182455	CC	33 (45.83%)	108.97 ± 13.89	82.58 ± 13.87	48.82 ± 7.27	29.15 ± 3.99	10.45 ± 1.60	11.82 ± 0.58	0.52 ± 0.76
	CT	36 (50.00%)	105.86 ± 13.49	75.61 ± 11.76	46.44 ± 9.78	27.50 ± 3.62	9.72 ± 2.15	11.58 ± 0.73	0.56 ± 1.00
	TT	3 (4.17%)	103.33 ± 24.42	76.67 ± 4.16	53.33 ± 4.04	29.33 ± 3.06	10.67 ± 0.58	12.00 ± 0.00	0.00 ± 0.00
	*F*		0.534	2.659	1.309	1.749	1.449	1.414	0.555
	*p *value		0.588	0.077	0.277	0.182	0.242	0.250	0.577
Dominant	CC	33 (45.83%)	108.97 ± 13.89	82.58 ± 13.87	48.82 ± 7.27	29.15 ± 3.99	10.45 ± 1.60	11.82 ± 0.58	0.52 ± 0.76
Model	CT+TT	39 (54.17%)	105.67 ± 14.12	75.69 ± 11.33	46.97 ± 9.61	27.64 ± 3.58	9.79 ± 2.08	11.62 ± 0.71	0.51 ± 0.97
	*t*		0.996	2.318	0.905	1.692	1.486	1.328	0.011
	*p *value		0.323	0.023	0.369	0.095	0.142	0.188	0.991
Recessive	CT+CC	69 (95.83%)	107.35 ± 13.67	78.94 ± 13.19	47.58 ± 8.69	28.29 ± 3.87	10.07 ± 1.93	11.70 ± 0.67	0.54 ± 0.88
Model	TT	3 (4.17%)	103.33 ± 24.42	76.67 ± 4.16	53.33 ± 4.04	29.33 ± 3.06	10.67 ± 0.58	12.00 ± 0.00	0.00 ± 0.00
	*t*		0.483	0.296	–1.136	–0.460	–0.530	–0.780	1.043
	*p *value		0.631	0.768	0.260	0.647	0.598	0.438	0.300

**Table 3.** **Effects of *Stmn1* gene polymorphism on SCWT and HVLT-R scores in males**.

SNP	Genotype	Total N (%)	SCWT			HVLT-R			
			SW	SC	SCW	TR	DR	TP	FP
rs182455	CC	23 (40.35%)	102.09 ± 15.48	75.48 ± 11.64	47.48 ± 9.88	27.43 ± 3.99	9.91 ± 1.91	11.65 ± 0.57	0.52 ± 0.67
	CT	29 (50.88%)	105.38 ± 17.71	72.90 ± 17.38	45.90 ± 10.53	25.83 ± 3.56	9.45 ± 1.62	11.38 ± 0.68	0.45 ± 0.74
	TT	5 (8.77%)	114.60 ± 13.48	64.80 ± 10.57	38.80 ± 7.16	27.80 ± 4.09	9.60 ± 1.52	11.80 ± 0.45	0.40 ± 0.55
	*F*		1.204	1.079	1.531	1.415	0.463	1.769	0.102
	*p *value		0.308	0.347	0.226	0.252	0.632	0.180	0.903
Dominant	CC	23 (40.35%)	102.09 ± 15.48	75.48 ± 11.64	47.48 ± 9.88	27.43 ± 3.99	9.91 ± 1.91	11.65 ± 0.57	0.52 ± 0.67
Model	CT+TT	34 (59.65%)	106.74 ± 17.30	71.71 ± 16.68	44.85 ± 10.33	26.12 ± 3.64	9.47 ± 1.58	11.44 ± 0.66	0.44 ± 0.71
	*t*		–1.038	0.940	0.958	1.290	0.954	1.247	0.433
	*p *value		0.304	0.351	0.342	0.203	0.344	0.218	0.667
Recessive	CT+CC	52 (91.23%)	103.92 ± 16.68	74.04 ± 15.03	46.60 ± 10.18	26.54 ± 3.80	9.65 ± 1.75	11.50 ± 0.64	0.48 ± 0.70
Model	TT	5 (8.77%)	114.60 ± 13.48	64.80 ± 10.57	38.80 ± 7.16	27.80 ± 4.09	9.60 ± 1.52	11.80 ± 0.45	0.40 ± 0.55
	*t*		–1.385	1.338	1.667	–0.705	0.066	–1.370	0.250
	*p *value		0.172	0.187	0.101	0.484	0.947	0.222	0.803

### 3.5 Sex Differences in Stmn1 Genotype Distribution and Cognitive Function 

The rs182455 genotype distribution did not differ between male and female participants (χ^2^ =1.313, *p *= 0.519). Significant sex differences were found for SC scores on the SCWT and TR scores on the HVLT-R (Table 4, t = –2.294, –2.490; *p* = 0.023, 0.014, respectively), but not for the remaining five SCWT and HVLT-R scores (Table 4, all *p* > 0.05).

**Table 4.** **Comparison of SCWT and HVLT-R scores between male and female participants with the same genotype**.

Neurocognitive Assessment		Variable scores (Mean ± SD)		t	*p*
		Male (N)	Female (N)		
SCWT	SW with 3 genotypes	104.86 ± 16.60 (57)	107.18 ± 14.02 (72)	–0.861	0.391
	SW with CC genotype	102.09 ± 15.48 (23)	108.97 ± 13.89 (33)	–1.740	0.087
	SW with CT genotype	105.38 ± 17.71 (29)	105.86 ± 13.49 (36)	–0.125	0.901
	SW with TT genotype	114.60 ± 13.48 (5)	103.33 ± 24.42 (3)	–0.862	0.422
	SC with 3 genotypes	73.23 ± 14.85 (57)	78.85 ± 12.93 (72)	–2.294	0.023
	SC with CC genotype	75.48 ± 11.64 (23)	82.58 ± 13.87 (33)	–2.009	0.050
	SC with CT genotype	72.90 ± 17.38 (29)	75.61 ± 11.76 (36)	–0.749	0.457
	SC with TT genotype	64.80 ± 10.57 (5)	76.67 ± 4.16 (3)	–1.814	0.120
	SCW with 3 genotypes	45.91 ± 10.15 (57)	47.82 ± 8.61 (72)	–1.155	0.250
	SCW with CC genotype	47.48 ± 9.88 (23)	48.82 ± 7.27 (33)	–0.585	0.561
	SCW with CT genotype	45.90 ± 10.53 (29)	46.44 ± 9.78 (36)	–0.217	0.829
	SCW with TT genotype	38.80 ± 7.16 (5)	53.33 ± 4.04 (3)	–3.163	0.019
HVLT-R	TR with 3 genotypes	26.65 ± 3.81 (57)	28.33 ± 3.82 (72)	–2.490	0.014
	TR with CC genotype	27.43 ± 3.99 (23)	29.15 ± 3.99 (33)	–1.584	0.119
	TR with CT genotype	25.83 ± 3.56 (29)	27.50 ± 3.62 (36)	–1.866	0.067
	TR with TT genotype	27.80 ± 4.09 (5)	29.33 ± 3.06 (3)	–0.556	0.598
	DR with 3 genotypes	9.65 ± 1.72 (57)	10.10 ± 1.89 (72)	–1.391	0.167
	DR with CC genotype	9.91 ± 1.91 (23)	10.45 ± 1.60 (33)	–1.151	0.255
	DR with CT genotype	9.45 ± 1.62 (29)	9.72 ± 2.15 (36)	–0.569	0.571
	DR with TT genotype	9.60 ± 1.52 (5)	10.67 ± 0.58 (3)	–1.139	0.298
	TP with 3 genotypes	11.53 ± 0.63 (57)	11.71 ± 0.66 (72)	–1.588	0.115
	TP with CC genotype	11.65 ± 0.57 (23)	11.82 ± 0.58 (33)	–1.055	0.296
	TP with CT genotype	11.38 ± 0.68 (29)	11.58 ± 0.73 (36)	–1.155	0.252
	TP with TT genotype	11.80 ± 0.45 (5)	12.00 ± 0.00 (3)	–0.750	0.482
	FP with 3 genotypes	0.47 ± 0.68 (57)	0.51 ± 0.87 (72)	–0.285	0.776
	FP with CC genotype	0.52 ± 0.67 (23)	0.52 ± 0.76 (33)	0.034	0.973
	FP with CT genotype	0.45 ± 0.74 (29)	0.56 ± 1.00 (36)	–0.482	0.631
	FP with TT genotype	0.40 ± 0.55 (5)	0.00 ± 0.00 (3)	1.633	0.178

SD, standard deviation.

Subsequently, we compared SCWT and HVLT-R scores between males and females with the same genotype. Significant or marginally significant sex differences were found for SC scores on the SCWT for the CC genotype and SCW scores on the SCWT for the TT genotype (Table 4, t = –2.009, –3.163; *p* = 0.050, 0.019, respectively), which are both consistent (SC) and different (SCW) from the results of the above comparisons of SCWT and HVLT-R scores between males and females with all genotypes. Since only five males and three females carried the TT genotype in the present study, the significance of the differences in SCW scores between males and females carrying the TT genotype could not be determined because of the small sample size.

## 4. Discussion 

The present study investigated the association between *Stmn1* gene polymorphism and cognitive function assessed by the SCWT and HVLT-R in a Chinese population for the first time. The results showed significant effects of the SNP rs182455 CC: (CT+TT) genotype on the SC score of the SCWT and TR score of the HVLT-R in the total sample, indicating that carriers of the rs182455 CC genotype have higher immediate free recall, cognitive flexibility, and ability to inhibit usual responses.

In the last decade, meta-analyses of genome-wide association studies (GWAS) have established that human general cognitive ability is highly heritable and polygenic [10, 34, 35, 36, 37, 38, 39, 40]. Prior studies focused on general cognitive ability, namely intelligence, and identified some loci, genes, and SNPs significantly associated with intelligence. Davies *et al*. [40] identified 148 independent loci associated with general cognitive function by GWAS and 709 genes associated with general cognitive function using gene-based analyses. Up to 4.3% of the variance in general cognitive function could be predicted by polygenic scores in independent samples [40]. However, *Stmn1* was not one of the genes associated with general cognitive ability in previous studies.

Stmn1 was the first discovered member of the stathmin family of phylogenetically related MT-destabilizing phosphoproteins, which also includes superior cervical ganglia neural-specific 10 protein (SCG10; stathmin 2), SCG10-like protein (SCLIP; stathmin 3), and RB3 (stathmin 4). Among these, Stmn1 is the most abundantly expressed in the nervous system [41]. Stmn1, a 19 kDa cytosolic protein, is composed of 149 amino acids organized into four domains defined by limited proteolysis [42]. MTs are protein polymers composed of α/β tubulin heterodimers with essential roles in cellular structure and function, including cell motility, polarity and proliferation, intracellular transport, neurite branch growth, and synaptic plasticity [4, 43, 44, 45]. Unphosphorylated or hypophosphorylated Stmn1 interacts with tubulin heterodimers to prevent the formation of MTs [46]. Stmn1 has four phosphorylation domains: Ser 16, 25, 38 and 63 [47]. Stmn1 releases tubulin after phosphorylation, so that MT can be formed. Thus, Stmin1 is involved in MT dynamics by regulating both the formation of MT and disassembly [44] and influences neural functions. Based on these functions, researchers have investigated the impact of Stmn1 on neural function in both experimental animals and humans.

LTP is an important form of synaptic plasticity [48, 49] and is regarded as a neuronal correlate of fear, learning, and memory [50, 51]. In 2005, Shumyatsky *et al*. [21] reported that whole-cell recordings from amygdala slices isolated from *Stmn1* knockout (KO) mice showed deficits in spike-timing-dependent LTP. Moreover, *Stmn1* KO mice exhibited decreased amygdala-dependent fear conditioning memory and failed to recognize the danger in innately aversive environments [21]. In 2008, Martel *et al*. [52] reported that *Stmn1* KO mice showed an increase in social interactions but a decrease in social recognition memory and affiliative maternal care, which were associated with* Stmn1* expression in the basolateral amygdala. In 2014, Uchida *et al*. [53] demonstrated that *Stmn1* transgenic mice expressing Stat4A, an unphosphorylatable *Stmn1* mutant, showed reduced contextual fear memory, and that Stmn1-MT interactions were a possible mechanism underlying age-dependent memory loss. In 2019, Nguyen *et al*. [54] found that *Stmn1* KO mice showed anxious hyperactivity, impaired object recognition, and decreased levels of neutral and social investigating behaviors at baseline. In a recent study, Shan *et al*. [55] demonstrated that Stmn1 in the brain memory loop, especially the hippocampus, regulated MT structure by phosphorylation at Ser 25 and Ser 38, and was thus involved in the mediation of fear memory abnormalities in post-traumatic stress disorder (PTSD). Therefore, animal experiments support a correlation between Stmn1 and cognitive functions like learning and memory, as well as related emotions like fear.

In contrast to the animal literature, studies on *Stmn1* gene function in humans are rare. Brocke *et al*. [24] reported that two *Stmn1*SNPs (rs182455 and rs213641) had significant effects on fear and anxiety responses in humans, measured by startle and cortisol stress responses. Cao *et al*. [56] found that the *Stmn1* rs182455 genotype significantly predicted the severity of reexperiencing symptoms in female patients with PTSD. There is only one report of a direct correlation between *Stmn1* gene polymorphism and cognitive function in humans. Ehlis *et al*. [23] found altered cognitive-affective processing in carriers of the rs182455 SNP C allele, as probed using three experimental paradigms. Of note, the SNP rs182455 selected in the present study is consistent with the SNP studied in the three previous studies [23, 24, 56]. In terms of the correlation between *Stmn1* variation and human cognitive function, our findings support the previous results [23]. However, the studies differ in several ways. First, different cognitive assessment tools were used in the study by Ehlis *et al*. [23] and the present study, and thus different aspects of cognitive functions were studied. The previous study mainly focused on emotion regulation, whereas the present study evaluated individual ability to inhibit habitual responses and react selectively to information, as well as verbal learning and memory. Second, the samples varied in terms of race and size: 59 healthy German participants were used by Ehlis *et al*. [23], whereas the present study involved 129 healthy Han Chinese participants. Therefore, our findings of a direct relationship between *Stmn1* gene variants and cognitive function assessed by the SCWT and HVLT-R in a Chinese sample are a confirmation and extension of previous findings.

The present study found no significant differences in SW and SCW scores on the SCWT or DR, TP and FP scores on the HVLT-R between males and females. However, SC scores on the SCWT and TR scores on the HVLT-R were significantly higher for females than for males, indicating that females have higher immediate free recall, cognitive flexibility, and ability to inhibit usual responses than males. Numerous studies have reported significant sex differences in cognitive function in different groups [57, 58, 59, 60]. Some reviews also summarized and addressed such sex specificity for cognitive functions in detail [61, 62]. Therefore, the findings of the present study that two dimensions of cognitive assessment differed between males and females are consistent with the conclusions of previous related research.

The present study also demonstrated a significant female-specific difference in the SC dimension of the SCWT among the rs182455 genotype groups (females carrying the CC genotype had significantly higher SC scores than those with the TT and CT genotypes). Further analysis showed that the difference in SC dimensions between the two sexes might derive mainly from differences in SC scores between the two sexes with the rs182455 CC genotype. This suggests that the interaction between *Stmn1* variants (especially the rs182455 CC genotype) and sex could influence an individual’s cognitive function. In previous studies, Brocke *et al*. [24] found that the *Stmn1* rs182455 genotype interacted with sex to significantly impact fear and anxiety responses, Cao *et al*. [56] found that the rs182455 C allele was only significantly associated with reexperiencing PTSD symptoms in females, and Ehlis *et al*. [23] found that the effects of the rs182455 C allele appeared more significant on altered cognitive–affective processing in females. Thus, the female-specific results on the effect of the rs182455 CC genotype on cognitive function in the present study thus support and build upon previous findings.

We speculate why SC scores were higher in females than in males and why the rs182455 CC genotype was significantly associated with SC scores in females, but not in males. As Cahill pointed out, sex differences at the level of brain organization and function are neither small nor unreliable [63]. Given the large amount of evidence that males and females differ in terms of brain regions involved in emotional regulation and cognition, as well as in several neurotransmitter systems, the influence of sex as an additional factor—as well as a factor interacting with other variables—must be considered. Another potential reason for sex-specific findings is androgens secreted by the ovaries of women, which might be involved in MT dynamics that affect neurite branch growth and synaptic plasticity, ultimately affecting biological determination of cognitive function. Regarding the observed sex effects, *in silico* analysis has demonstrated the transcriptional influence of rs182455 with the T allele facilitating the binding of estrogen regulated transcription factors [23]. Therefore, the interaction between sex and *Stmn1* SNP genotypes might be the underlying mechanism of individual differences in cognitive ability. However, this speculation needs to be confirmed by further biochemical experiments.

In summary, this study investigated associations between *Stmn1* rs182455 polymorphisms and cognitive abilities measured by the SCWT and HVLT-R in a sample of healthy Han Chinese individuals, demonstrating the underlying biogenetic mechanisms regulating cognition. The results demonstrated a significant modulating influence of SNP rs182455 on the SC dimension of the SCWT and the TR dimension of the HVLT-R, indicating that individuals with the rs182455 CC genotype have higher immediate free recall and cognitive flexibility than those with the CT or TT genotypes. A significant difference in the SC dimension of the SCWT was also observed for the rs182455 genotype in females, indicating that this association is sex-specific. These results provide supporting evidence that *Stmn1* gene variations and sex impact cognitive abilities in healthy Chinese individuals and may contribute to the etiopathogenesis of many neurodegenerative and psychiatric conditions since cognitive impairment is their core symptom. Moreover, the Stmn1 protein has been known to play an important role in neurite extension and growth, which typically occur during neural development, repair, and regeneration. Next, further studies, including experiments with gene knockout animals, should be conducted to determine the exact and specific role of the *Stmn1* gene and protein in cognitive function. The results of the present study and further studies will provide empirical evidence for the abnormalities of neurodevelopment and synaptic plasticity in the pathogenesis of many neuropsychiatric disorders with cognitive impairment and provide indicators and an evidence-based basis for the prevention and treatment of such disorders in the future.

However, the results of the present study should be viewed with caution because of the limited sample size and age range, the limited number of studied SNPs, and the limitations of the neurocognitive assessment tools used, which might increase the potential bias. Moreover, the present study design was a cross-sectional observational study. This design can only describe the correlation between two variables, not directly prove causation, and it is difficult for this design to completely exclude the effects of confounding factors, which may mask or exaggerate the true association between two variables. In the future, studies of rs182455 SNP and other *Stmn1* gene SNPs in larger Chinese or other ethnic samples, as well as a cohort study design, are needed to verify these findings. Research on the specific mechanisms underlying the influence of *Stmn1* variants on individual cognitive function is also needed to develop a more comprehensive understanding of this effect.

## 5. Conclusions

All in all, the present findings suggest that rs182455 *Stmn1* polymorphism might affect cognitive flexibility and immediate free recall in healthy Chinese individuals, especially females.

## Availability of Data and Materials

The raw data supporting the results and conclusions of this article are available upon request.
